# Experimental Cannabinoid 2 Receptor Activation by Phyto-Derived and Synthetic Cannabinoid Ligands in LPS-Induced Interstitial Cystitis in Mice

**DOI:** 10.3390/molecules24234239

**Published:** 2019-11-21

**Authors:** Geraint Berger, Nipun Arora, Ian Burkovskiy, Yanfang Xia, Anu Chinnadurai, Robert Westhofen, Georg Hagn, Ashley Cox, Melanie Kelly, Juan Zhou, Christian Lehmann

**Affiliations:** 1 Department of Anesthesia, Pain Management and Perioperative Medicine, Dalhousie University, Halifax, NS B3H 4R2, Canada; gcberger@dal.ca (G.B.); np564125@dal.ca (N.A.); ian.burkovskiy@gmail.com (I.B.); xia.yf@foxmail.com (Y.X.); 13ac123@queensu.ca (A.C.); robert.westhofen@stud.pmu.ac.at (R.W.); georg.hagn@stud.pmu.ac.at (G.H.); melanie.kelly@dal.ca (M.K.); juan.zhou@dal.ca (J.Z.); 2 Department of Pharmacology, Dalhousie University, Halifax, NS B3H 4R2, Canada; 3 Department of Microbiology & Immunology, Dalhousie University, Halifax, NS B3H 4R2, Canada; 4 Department of Urology, Dalhousie University, Halifax, NS B3H 4R2, Canada; ashleycox1000@gmail.com; 5 Department of Ophthalmology & Visual Sciences, Dalhousie University, Halifax, NS B3H 4R2, Canada

**Keywords:** interstitial cystitis, endocannabinoid system, sesquiterpenoid, inflammation, cannabinoid receptors, allodynia, microcirculation

## Abstract

Interstitial cystitis (IC) is a chronic bladder disorder with unclear etiology. The endocannabinoid system has been identified as a key regulator of immune function, with experimental evidence for the involvement of cannabinoid receptors in bladder inflammation. This study used intravital microscopy (IVM) and behavioral testing in lipopolysaccharide-induced IC, to investigate the anti-inflammatory analgesic effects of a natural dietary sesquiterpenoid, beta-caryophyllene (BCP), which is present in cannabis among other plants, and has reported agonist actions at the cannabinoid 2 receptor (CB_2_R). BCP’s anti-inflammatory actions were compared to the synthetic CB_2_R-selective cannabinoid, HU308, and to an FDA-approved clinical treatment (dimethyl sulfoxide: DMSO). IVM data revealed that intravesical instillation of BCP and/or HU308 significantly reduces the number of adhering leukocytes in submucosal bladder venules and improves bladder capillary perfusion. The effects of BCP were found to be comparable to that of the selective CB_2_R synthetic cannabinoid, HU308, and superior to intravesical DMSO treatment. Oral treatment with BCP was also able to reduce bladder inflammation and significantly reduced mechanical allodynia in experimental IC. Based on our findings, we believe that CB_2_R activation may represent a viable therapeutic target for IC, and that drugs that activate CB_2_R, such as the generally regarded as safe (GRAS) dietary sesquiterpenoid, BCP, may serve as an adjunct and/or alternative treatment option for alleviating symptoms of inflammation and pain in the management of IC.

## 1. Introduction

Interstitial cystitis (IC) is a chronic inflammatory disorder of the urinary bladder, defined by the Society for Urodynamics and Female Urology (SUFU) as “an unpleasant sensation perceived to be related to the urinary bladder, associated with lower urinary tract symptoms for more than six weeks duration, in the absence of infection or other identifiable causes” [1]. The main symptoms of IC include chronic pelvic pain and urinary symptoms, such as persistent urge to void and/or urinary frequency [2]. A managed care population study concluded that the prevalence and incidence of IC is higher in women than men. The male-to-female ratio is estimated to be 5:1 [3]. While IC is recognized as a disease causing a profound negative effect on patients’ quality of life, to-date no definite etiologies have been identified and therefore, a curative treatment remains elusive. Nonetheless, the three most common proposed etiologies for IC in the literature are bladder urothelial dysfunction, neurogenic inflammation, and neuropathic pain [4,5].

Given the unclear pathophysiology of IC, current treatments are aimed towards reduction of inflammation and pain relief. The most common approaches to therapy are oral medications and bladder instillations. Commonly used oral therapies include pentosan polysulfate (PPS) and tricyclic antidepressants, which have been found to be beneficial in relieving symptoms through various mechanisms [6]. Intravesical therapies, where the pharmacological agent is delivered directly into the bladder, have provided relief to some patients. Dimethyl sulfoxide (DMSO) is one such pharmacological agent that is FDA approved for IC (RIMSO-50^®^), and has been shown to have anti-inflammatory and analgesic effects [6]. Unfortunately, DMSO treatment relapse is not uncommon, and DMSO treatment has also shown to fail in patients with severe IC [7,8]. In addition to treatment failure, the long-term efficacy of DMSO treatment has yet to be properly studied, with follow up times of only 1–2 years long, it can be distressing for patients in the event that they experience a relapse later on in treatment [9,10]. Likewise, treatments with PPS and tricyclic anti-depressants are not promising either. One study revealed that PPS treatment’s effect was limited to only 32% of patients [11]. To this day, none of the commonly used medications for IC have been shown to completely alleviate pain and reduce inflammation of the bladder. The unmet need of an effective therapy for IC calls for novel approaches to help manage this debilitating disorder. 

The endogenous cannabinoid system (ECS) is involved in a variety of physiological processes including metabolism, pain-sensation, neurotransmission and inflammation [12,13]. The ECS consists of cannabinoid receptors, endogenous cannabinoid ligands, and endocannabinoid metabolizing enzymes. The most common endogenous cannabinoid ligands, or endocannabinoids, are *N*-arachidonylethanolamine or anandamide (AEA) and 2 arachidonoylglycerol (2-AG). These endocannabinoids are rapidly synthesized *de novo* from postsynaptic membrane-lipid precursors in the cell membrane [14]. Since the discovery of cannabinoid receptors and their associated endogenous and phytocannabinoid ligands, several synthetic ligands have been developed that selectively bind to the cannabinoid receptors [15]. The effects of the ECS are mediated by two cannabinoid receptors; cannabinoid receptor 1 (CB_2_R) and 2 (CB_2_R), both of which are G-coupled protein receptors that couple to G_i/o_ proteins upon activation to induce various signal transduction pathways [16]. Both AEA and 2-AG have higher affinities for CB_1_R over CB_2_R, but 2-AG also binds CB_2_R with high affinity [17]. While CB_1_R receptors are predominately expressed in the central nervous system (CNS) and in some peripheral tissues [18], CB_2_R are highly expressed on immune cells but have also been identified in select areas of the CNS and peripheral tissues, including the intestine and bladder [19,20]. Unlike CB_1_R, activation of CB_2_R by phyto-derived and synthetic cannabinoid ligands is not associated with psychotropic effects, and has demonstrated immunomodulatory and anti-inflammatory actions [15]. More specifically, CB_2_R agonists have been shown to inhibit the production of pro-inflammatory mediators and decrease neutrophil chemotaxis and extravasation [21,22]. The presence of CB_2_R and CB_1_R in sensory nerves of the detrusor and mucosa (urothelium and suburothelium) of human bladders [23] suggests that modulation of the ECS may represent a potential pharmacological target for IC therapy.

**Figure 1:**
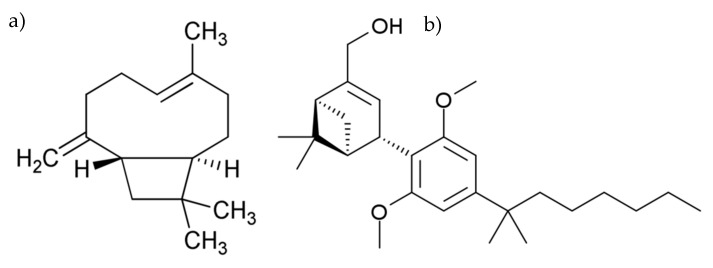
Chemical structures of (**a**) Beta-Caryophyllene and (**b**) HU308.

The aim of this study was to investigate the therapeutic potential of CB_2_R activation to reduce inflammation and pain in experimental IC using beta-caryophyllene (BCP), a sesquiterpenoid found in cannabis and other plant sources that has been reported to act as a CB_2_R agonist (BCP, K_i_ = 155 ± 4 nM [24], Figure 1a), versus a selective synthetic CB_2_R agonist, HU308 (K_i_ = 22.7 ± 3.9 nM [25], Figure 1b). Experimental IC was induced either via systemic (intraperitoneal, i.p.) or local (intravesical) lipopolysaccharide (LPS) administration. With respect to the latter model, the effectiveness of both intravesical and oral BCP administration was evaluated against that of an FDA approved clinical treatment for IC, intravesical instillation of RIMSO-50^®^ (DMSO 50% *w/w* aqueous solution). Given the importance and selectivity of the CB_2_R pathway in modulating the immune system, we hypothesized that activation of local CB_2_R signalling during LPS-induced cystitis will reduce pain and the degree of inflammation in experimental IC

## 2. Results

**Figure 2:**
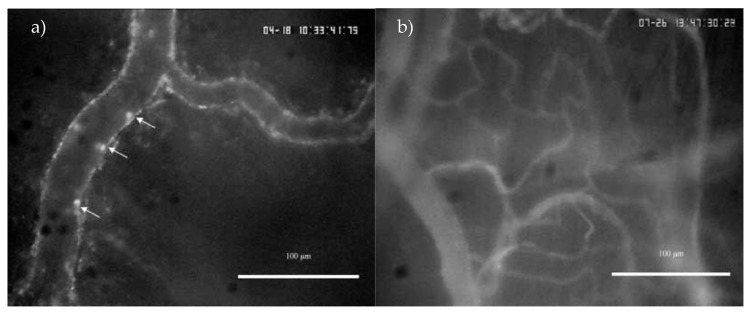
Still frame images from leukocyte trafficking (**a**) and capillary perfusion (**b**) videos obtained from intravital microscopy of mouse bladder (magnification = 200×). Rhodamine-6G and FITC were administered i.v into the tail vein to allow fluorescent visualization of leukocyte-endothelial interactions and capillary blood flow, respectively. White arrows indicate leukocytes interacting with the endothelium of the venule.

Representative still frame images from leukocyte trafficking and capillary perfusion videos obtained from intravital microscopy of the bladder are shown in Figure 2. 

### 2.1. IC Induced by i.p. LPS Administration

**Figure 3:**
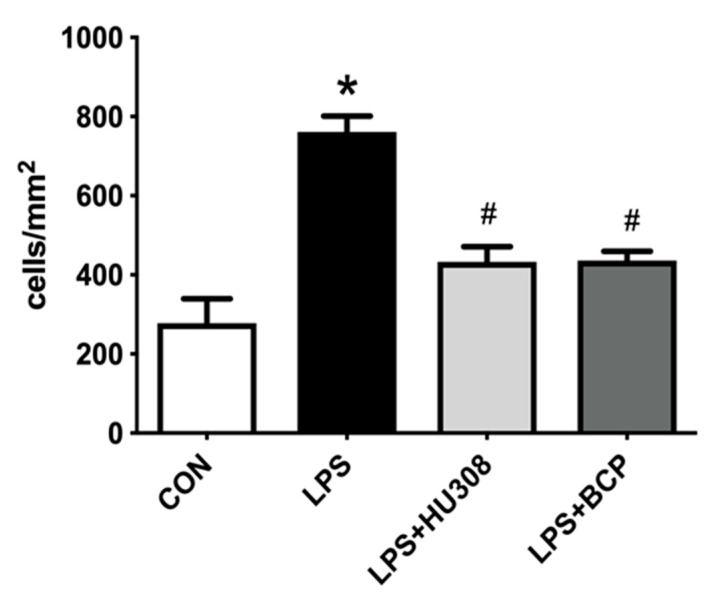
Leukocyte adhesion in submucosal bladder venules of female CD-1 mice for the following experimental groups: control (CON; *n* = 9), lipopolysaccharide (LPS)-induced interstitial cystitis (IC) (LPS; *n* = 9), LPS-induced IC treated with HU308 (5 mg/kg, LPS + HU308; *n* = 4), LPS-induced IC treated with beta-caryophyllene (BCP) (100 mg/kg, LPS + BCP; *n* = 7). LPS, BCP, and HU308 were all administered via i.p. injection. Data presented as mean ± SD. * *p* < 0.05 vs. CON. # *p* < 0.05 vs. LPS.

Systemically (i.p) administered LPS significantly increased the number of leukocytes adhering to the endothelium of submucosal bladder venules. Treatment with BCP (100 mg/kg) and HU308 (5 mg/kg) significantly reduced the number of adherent leukocytes in LPS challenged animals. No difference in the number of adherent leukocytes was observed between the BCP and HU308 treatment groups (Figure 3).

**Figure 4:**
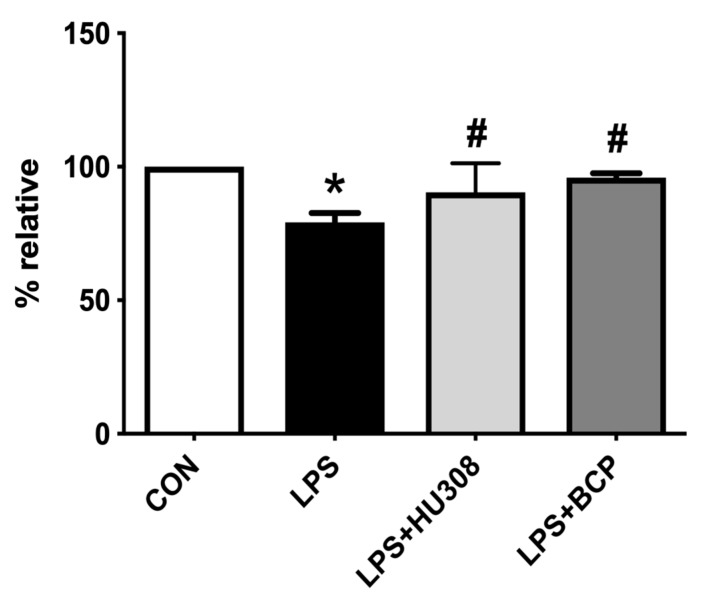
Capillary perfusion quantified through functional capillary density (FCD) within the bladder microcirculation of female CD-1 mice for the following experimental groups: control (CON; *n* = 9), lipopolysaccharide (LPS)-induced IC (LPS; *n* = 9), LPS-induced IC treated with HU308 (5 mg/kg, LPS + HU308; *n* = 4), LPS-induced IC treated with BCP (100 mg/kg, LPS + BCP; *n* = 7). Saline, LPS, BCP, and HU308 were all administered via intraperitoneal injection. Data are presented as percentage relative to the control group. * *p* < 0.05 vs. CON. # *p* < 0.05 vs. LPS.

The functional capillary density (FCD) of the bladder microcirculation was significantly reduced following LPS administration (Figure 4). Treatment with BCP, as well as with HU308 restored capillary perfusion to levels observed in healthy control animals. There was no significant difference in functional capillary density between the BCP and HU308 treatment groups.

### 2.2. IC Induced by Intravesical LPS Administration

**Figure 5:**
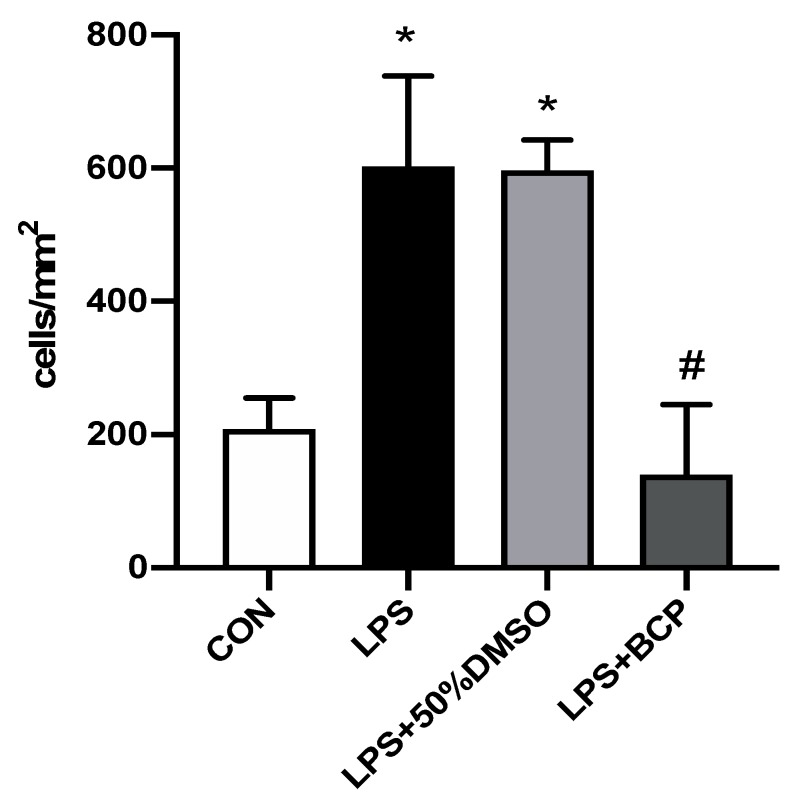
Leukocyte adhesion in submucosal bladder venules of female BALB/c mice for the following experimental groups: control (CON; *n* = 6), LPS-induced IC (LPS; *n* = 5), LPS-induced IC treated with 50% DMSO (LPS + 50% DMSO; *n* = 5), and LPS-induced IC treated with BCP (100 mg/kg, LPS + BCP; *n* = 6). Saline, LPS, DMSO, and BCP were all administered via intravesical instillation. Data presented as mean ± SD. * *p* < 0.05 vs. CON. # *p* < 0.05 vs. LPS.

A low baseline level of leukocyte adherence was observed in control animals administered intravesical saline. A significant increase in the number of adherent leukocytes was observed in animals administered intravesical LPS compared to control animals. BCP treatment significantly decreased the number of adherent leukocytes to the degree observed in healthy control animals. In contrast, the 50% DMSO treatment retained a higher number of adherent leukocytes (Figure 5). 

**Figure 6:**
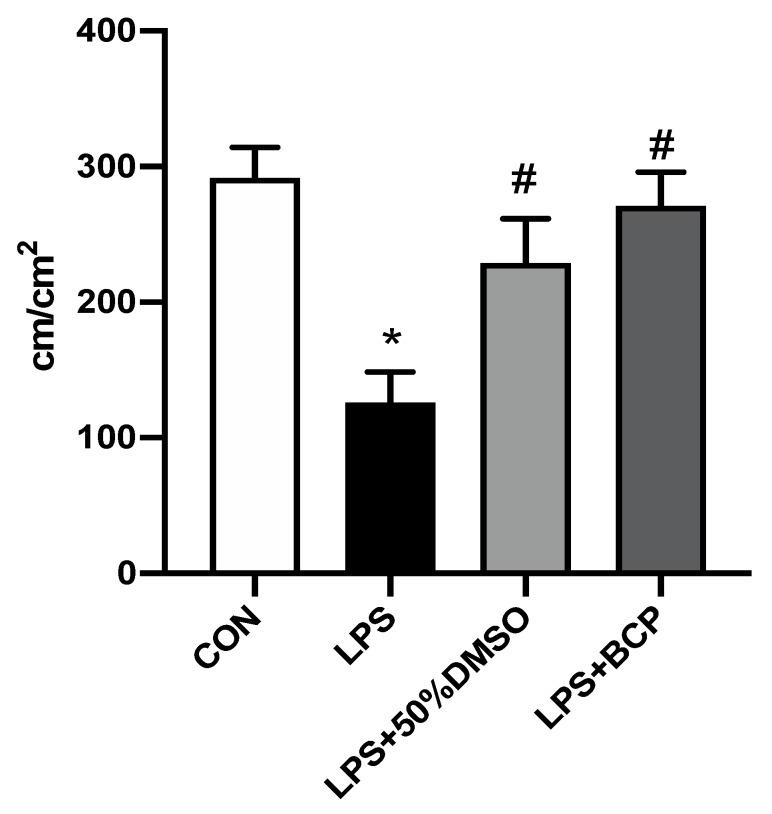
Capillary perfusion quantified through FCD within the bladder microcirculation of female BALB/c mice for the following experimental groups: control (CON; *n* = 6), LPS-induced IC (LPS; *n* = 5), LPS-induced IC treated with 50% DMSO, (LPS + 50% DMSO, *n* = 5), LPS-induced IC treated with BCP (100 mg/kg, LPS + BCP; *n* = 6). Saline, LPS, DMSO, and BCP were all administered via intravesical instillation. Data are presented as mean ± SD. * *p* < 0.05 vs. CON. # *p* < 0.05 vs. LPS.

Intravesical LPS administration significantly reduced the capillary perfusion as expressed by FCD in the bladder microcirculation. Administration of BCP restored perfusion to levels observed in healthy control animals. Treatment with intravesical DMSO also significantly improved capillary perfusion following LPS instillation (Figure 6). 

### 2.3. IC Induced by Intracesical LPS Administration Treated with Oral BCP

**Figure 7:**
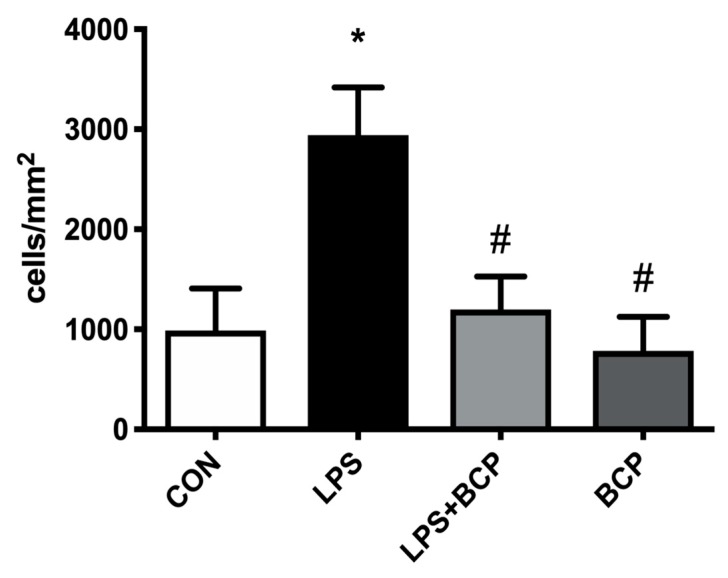
Leukocyte adhesion in submucosal bladder venules of female BALB/c mice for the following experimental groups: control (CON; *n* = 5), LPS-induced IC (LPS; *n* = 5), LPS-induced treated with oral BCP (100 mg/kg, LPS + BCP; *n* = 5), healthy animals administered oral BCP (100 mg/kg, BCP; *n* = 5). All groups were pretreated with olive oil gavage, with the treatment groups receiving BCP dissolved in olive oil. Saline and LPS were administered via intravesical instillation. Data presented as mean ± SD. * *p* < 0.05 vs. Control, # *p* < 0.05 vs. LPS.

As shown in Figure 7, untreated animals administered LPS via intravesical instillation showed a high level of leukocyte adherence in submucosal bladder venules. As seen with intravesical BCP instillation (Figure 5), a significant decrease in the number of adherent leukocytes was also observed in animals treated with orally administered BCP (100 mg/kg. Figure 7). Oral BCP treatment in healthy animals did not produce any significant changes in the number of adherent leukocytes relative to the control group.

**Figure 8:**
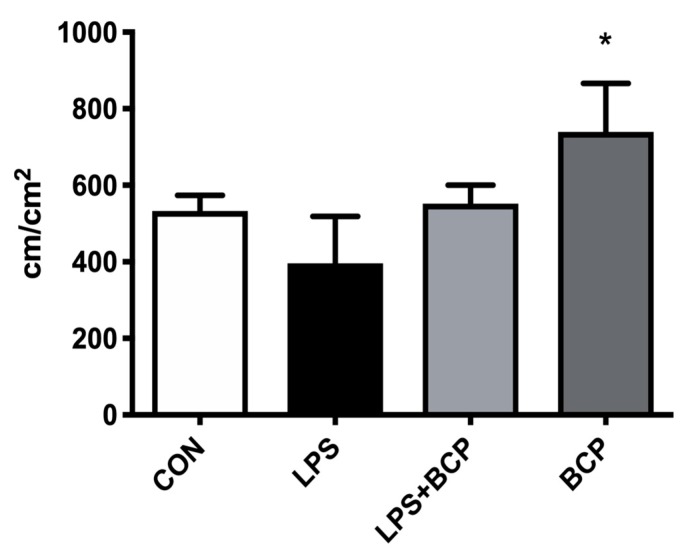
Capillary perfusion quantified through FCD within the bladder microcirculation of female BALB/c mice for the following groups: control (CON; *n* = 5), LPS-induced IC (LPS; *n* = 5), IC treated with oral BCP (100 mg/kg, LPS + BCP; *n* = 5), healthy animals administered oral BCP (100 mg/kg, BCP; *n* = 5). All groups were pretreated with olive oil gavage, with the treatment groups receiving BCP dissolved in olive oil. Saline and LPS were administered via intravesical instillation. Data presented as mean ± SD. * *p* < 0.05 vs. LPS.

No significant differences in FCD were observed between the untreated IC group and the group treated with oral BCP. There was a significant improvement in FCD in the experimental group treated only with BCP, when compared to the untreated IC group (Figure 8). 

### 2.4. Behavioral Analysis and Von Frey Aesthesiometry

**Figure 9:**
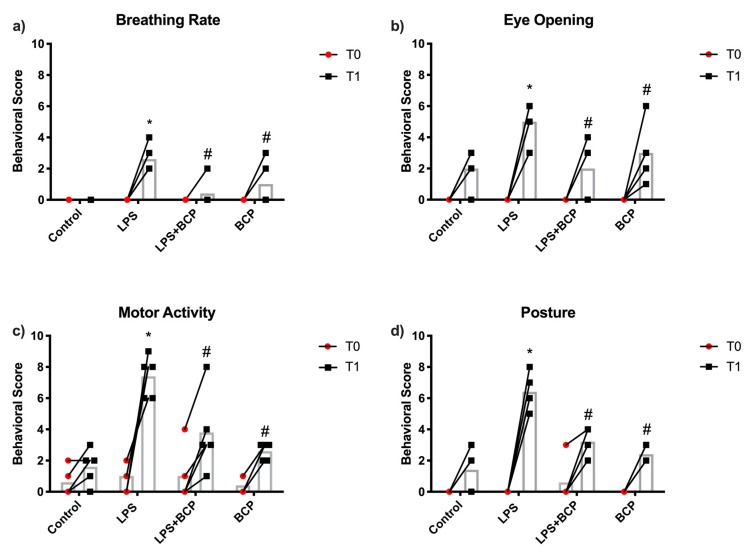
Behavioral score for each category of female BALB/c mice; (**a**) breathing rate, (**b**) eye opening, (**c**) motor activity, (**d**) posture, before cystitis induction (T0) and after treatment (T1) for the following experimental groups: healthy control animals (Control, *n* = 5), LPS-induced IC (LPS; *n* = 5), LPS-induced IC treated with oral BCP (100 mg/kg, LPS + BCP; *n* = 5), and healthy animals administered BCP (100 mg/kg, BCP; *n* = 5) All groups were pretreated with olive oil gavage, with the treatment groups receiving BCP dissolved in olive oil. Saline and LPS were administered via intravesical instillation. Data presented as the mean score ± SD for each parameter. Individual data points are also shown. * *p* < 0.05 vs. Control, # *p* < 0.05 vs. LPS.

To evaluate the pain induced by IC and the potential analgesic effects of BCP, behavioral analysis and von Frey aesthesiometry were performed. A significant increase in breathing rate was observed in animals with untreated LPS-induced IC. Breathing rate was significantly reduced in animals that were administered oral BCP compared to untreated animals. Similarly, animals with untreated LPS-induced IC scored significantly higher on eye -opening. This score was significantly reduced following oral BCP treatment. Furthermore, significant improvements in motor activity and posture were observed in animals treated with oral BCP (Figure 9).

**Figure 10:**
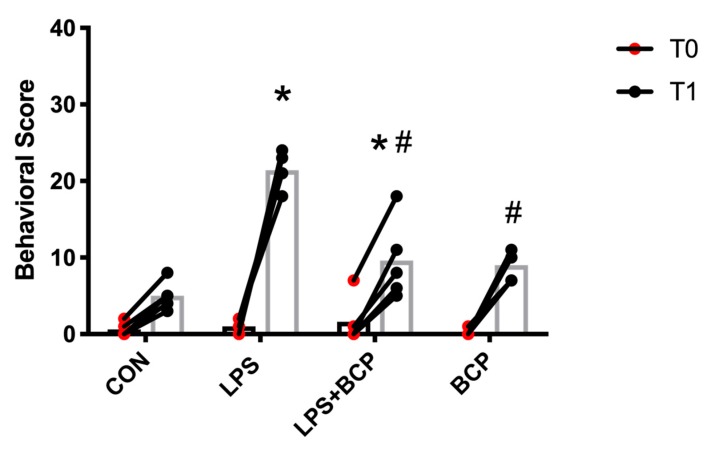
Composite score of behavioral results of female BALB/c mice before cystitis induction (T0) and after treatment (T1) for the following experimental groups: control (CON; *n* = 5), LPS-induced IC (LPS; *n* = 5), LPS-induced IC treated with BCP (100 mg/kg, LPS + BCP; *n* = 5), and BCP administered to healthy animals (100 mg/kg, BCP; *n* = 5). All groups were pretreated with olive oil gavage, with the treatment groups receiving BCP dissolved in olive oil. Saline and LPS were administered via intravesical instillation. Data presented as mean composite score ± SD. Individual data points are also shown. * *p* < 0.05 vs. Control, # *p* < 0.05 vs. LPS.

A significant increase in the cumulative behavioral score was observed in the untreated LPS-induced IC group compared to the control group at T1. In addition, the behavioral score at T1 of the IC group treated with oral BCP was significantly less than the untreated IC group. Treatment with oral BCP produced a significant reduction in the behavioral score, suggesting a reduction in the overall level of pain and discomfort (Figure 10).

**Figure 11:**
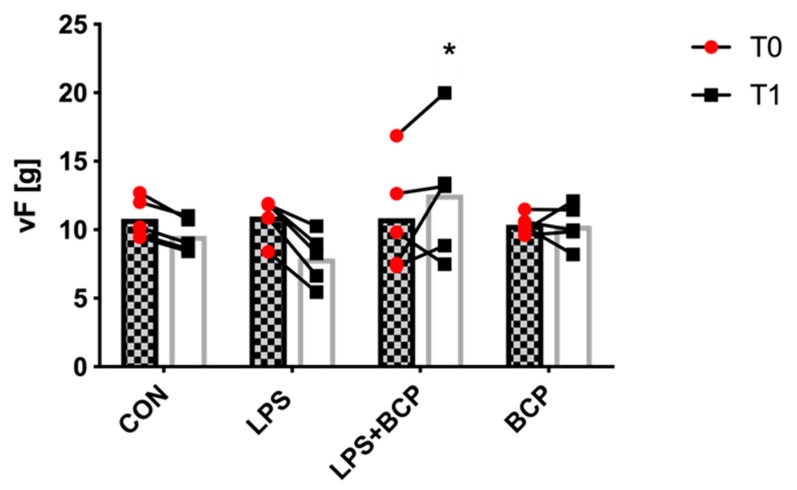
Force applied (g) by an electronic von Frey aesthesiometer before a response withdrawal was observed in female BALB/c mice for the following experimental groups: control (CON; *n* = 5), untreated LPS-induced IC (LPS; *n* = 5), LPS-induced IC treated with BCP (100 mg/kg, LPS + BCP; *n* = 5) and BCP administered to healthy animals (100 mg/kg, BCP; *n* = 5). All groups were pretreated with olive oil gavage, with the treat groups receiving BCP dissolved in olive oil. Saline and/or LPS was administered via intravesical instillation. Data presented as mean total force ± SD before cystitis induction (T0) and after the treatment period (T1). Individual data points are also shown. * *p* < 0.05 T1 LPS + BCP vs. T1 LPS.

A significant increase in applied force was recorded in animals with LPS-induced cystitis that were treated with oral BCP. BCP treatment produced a significant improvement in the withdrawal threshold, suggesting that BCP reduced levels of discomfort and pain following treatment (Figure 11).

## 3. Discussion

In summary, the results of this study showed the following: 1) i.p. BCP administration restored capillary perfusion and reduced leukocyte-endothelial adhesion to the same extent as the synthetic CB_2_R agonist HU308 in an IC model induced by systemic LPS administration; 2) intravesical BCP was more effective at reducing leukocyte-endothelial adhesion in submucosal bladder venules compared to intravesical DMSO, and significantly restored tissue perfusion to a greater extent than DMSO in a local LPS-induced model of IC; 3) Orally administered BCP significantly reduced the number of adherent leukocytes in submucosal venules in the same local model of IC. In addition, oral BCP was shown to provide analgesia for IC-induced pain. 

Beta-caryophyllene is a naturally occurring sesquiterpene found in many plants including cloves, black pepper and cannabis and is a common GRAS addictive approved by the FDA for use in food. BCP has been reported to selectively bind the CB_2_R, and because of structural differences from the classical phytocannabinoids (i.e., tetrahydrocannabinol, cannabidiol) found in the plant, BCP is often referred to as an “atypical cannabinoid” [26]. Given the GRAS status of BCP and the non-psychotropic, anti-inflammatory and analgesic actions reported for ligands that activate CB_2_R, BCP has potential for the management of symptoms of chronic inflammatory disease. In our study, BCP was examined for its anti-inflammatory activity in bladder inflammation and compared against the known CB_2_R agonist HU308. HU308 was selected due to its specificity for CB_2_R and anti-inflammatory actions reported in pre-clinical models. HU308 has been used to study the anti-inflammatory effects of CB_2_R signalling in a number of inflammatory conditions, including uveitis [27], periodontitis [28], and recently in Parkinson’s disease [29]. Leukocyte-endothelial adherence was significantly reduced in submucosal bladder venules in mice treated with BCP or HU308. There was no difference between the two treatment groups, indicating comparable efficacy between the two compounds in their ability to attenuate leukocyte adhesion in submucosal bladder venules following LPS challenge.

Our results are consistent with several studies that have demonstrated the anti-inflammatory effects of CB_2_R activation in the vasculature of various tissues, including the intestine, pia mater, and retina [27,30,31,32,33]. Tambaro et al. (2014) positively identified the protective role of CB_2_R in a model of LPS-induced IC, similar to the first model used in this study. They showed that systemic administration of JWH015 (CB_2_R agonist) decreased leukocyte infiltration and reduced the expression IL-1α, IL-1β, and TNF-α in the bladder. Tambaro et al. (2014) also demonstrated that the anti-inflammatory protection was solely due to the actions of JWH015. This was confirmed by administration of AM630 (CB_2_R antagonist) to reverse the actions of JWH015. In contrast to JWH015, intraperitoneal administration of the selective CB_1_R agonist arachidonyl-2′-chloroethylamide (ACEA, K_i_ = 1.4 nM) failed to show the protective effects of the JWH015 [34]. With respect to our primary study substance BCP, Gertsch et al. (2008) [26] demonstrated that BCP and its isomers do not show significant binding affinity for human CB_1_R. In addition to Tambaro et al. (2014), and to our knowledge, there have only been a few studies that have examined the involvement of the CB_2_R activation in experimental cystitis [35,36]. CB_2_R expression has been shown to be significantly increased in both chronically and acutely inflamed bladders [37]. Furthermore, in recent pilot experiments performed in our lab, we have observed worsening of IC pathology in CB_2_R knockout mice (Figure S1). These findings are consistent with Szczesniak et al. (2017) who reported that intraocular inflammation in experimental proliferative vitreoretinopathy was increased in CB_2_R knockout mice [38]. Taken together, these findings suggest that the changes in CB_2_R expression and receptor signalling following inflammation of the bladder may function to suppress the inflammatory response and associated pain, and support CB_2_R as a potential target for pharmacological IC therapy.

In addition to reducing leukocyte adhesion, we also observed significant improvements in microvascular bladder perfusion following i.p. and intravesical administration of BCP, as well as with DMSO treatment. Microvascular perfusion is an important indicator of the general physiological status of the tissue or organ. Intraperitoneal and intravesical LPS administration significantly reduces functional capillary density in the urinary bladder. Microcirculatory perfusion is regulated through the interplay of neuroendocrine, paracrine, and mechanosensory pathways [39]. During inflammation, a disruption in this process is noticed due to various factors including a decrease in the deformability of red blood cells, increased blood viscosity, and increased aggregability of activated leukocytes due to the upregulation of cellular adhesion molecules [39,40]. These mechanisms may account for the reduced FCD changes observed in our untreated experimental groups. Specifically, BCP and HU308′s direct or indirect inhibition of NF-κB, which is involved in increasing transcription and release of pro-inflammatory cytokines and adhesion molecules, may explain the improvements in perfusion observed in our study [27,41,42]. The significant improvement in vascular perfusion observed in the BCP and HU308 treatment groups, despite the excessive immunoactivation posed by LPS challenge, suggests a strong role of CB_2_R -related immunomodulation in experimental IC.

Although its mechanism is not fully understood, DMSO has served as one of the primary pharmacological approaches to treating IC since being approved by the FDA in 1978 [43]. Animal model studies have suggested that the anti-inflammatory activity of DMSO is related to inhibition of IL-8, decreased NF-κB activation, and prostaglandin E_2_ stimulation [43,44]. In this study, we show that intravesical BCP administration significantly reduced leukocyte adhesion and improved capillary perfusion to a greater extent than intravesical DMSO. Intravesical DMSO treatment has been met with mixed outcomes. A recent study investigating patient perception of success or failure of IC therpaies showed that only 36.9% of patients reported improved outcome following intravesical DMSO instillation [45]. DMSO instillation may also only be effective for IC patients with Hunners lesions [46], which has a prevalence ranging from 5–20% [47]. To our knowledge, this is the first time a natural phyto-derived cannabinoid receptor agonist has been tested intravesically for its ability to attenuate inflammation of the bladder. Intravesical drug delivery offers a number of potential benefits, including the ability to achieve a high drug concentration in the bladder, as well as minimize systemic side effects by reducing systemic drug exposure. However, low urothelial permeability and drug dilution caused by continuous urine production are inherent limitations to intravesical administration. Conversely, while oral therapeutics are often met with good patient compliance due to ease of administration, achieving an effective drug concentration in the bladder, while minimizing undesirable biodistribution, remains a challenge [48]. 

Oral administration of BCP has been previously studied in a number of animal models of inflammation. For example, oral pretreatment of BCP impaired Mycobacteirum bovis induced neutrophil accumulation in a mouse model of pleurisy [49]. BCP was also found to inhibit TNF-α induced expression of vascular cell adhesion protein 1 (VCAM-1), inhibiting macrophage infiltration to the aortic surface [50]. Inhibition of ICAM-1 with an anti-ICAM-1 antibody significantly reduced the degree of cyclophosphamide-induced severe non-bacterial cystitis in rats [51]. A similar effect was observed with BCP in a mouse model of cisplatin-induced renal inflammation, whereby pretreatment with oral BCP significantly attenuated renal ICAM-1 mRNA expression [42]. Our results, in combination with these findings, suggest that BCP may be a promising natural pharmacological agent for the management of inflammatory disorders, acting to attenuate leukocyte infiltration to the inflamed tissue by reducing endothelial adherence within the microcirculation. 

In our study, oral BCP treatment significantly reduced nociceptive behaviors following LPS administration, and significantly increased the amount of suprapubic force that could be applied to the supra-pubic region with von Frey filaments before a withdrawal response was observed. Our results are in accordance with a number of previous studies that have demonstrated the anti-nociceptive and analgesic effects of CB_2_R activation [52,53]. Intravenous administration of the CB_2_R agonists AM1241 and L768242 independently reduced the second phase of nocifensive behaviors induced by intraplanar formalin injection, and also reduced allodynia caused by spinal nerve ligation in a rat model of neuropathic pain [54]. In addition, CB_2_R agonists have also been shown to suppress thermal and mechanical hyperalgesia in rodents [55]. Oral administration of BCP was shown to reduce inflammatory pain responses in the formalin test, and also attenuate thermal hyperalgesia and mechanical allodynia [56]. In the same study, oral BCP was more effective at providing analgesia compared to subcutaneously administered JWH-113 (a synthetic CB_2_R agonist). Using an acrolein-induced model of cystitis, Wang et al. (2014) also showed that treatment with a CB_2_R agonist, GP1a, inhibited bladder inflammation associated peripheral mechanical sensitivity [36]. Together, these results support the potential use of CB_2_R agonists as possible therapies for the management of IC bladder pain. 

The current study examined the therapeutic effects of CB_2_R activation in two experimental models of IC, one induced via i.p. LPS administration, and the other via intravesical administration. One inherent limitation to this study is that the unclear etiology of IC makes this a difficult disorder to model in animals. Experimental IC has been induced via intravesical administration of a number of chemical irritants, including bacterial LPS, turpentine, mustard oil, acrolein, and cyclophosphamide [57]. In addition to irritant-induced cystitis, various models of autoimmune and neurogenic cystitis have also been used. The consensus of an extensive review of a number of currently used models of IC conducted by Bjorling, Wang, & Bushman (2011) was that none of the currently available animal models perfectly mimic clinical IC. Although these models may be beneficial in understanding the various mechanisms of bladder inflammation and pain, they are limited to demonstrating how the bladder responds to a noxious, often exogenous stimuli, and this is likely not fully representative of the complex pathophysiology behind IC [58]. In addition, many studies, including the present study, examine IC using an acute model of inflammation. Expanding the length of exposure to IC conditions in future studies may be beneficial in mirroring the clinical course of IC. Finally, potential variability of the immune status among patients and/or animals underscores the importance of accurate identification of the therapeutic window for CB_2_R treatment intervention, making it essential to establish a timeline when CB_2_R activation is the most effective. Analysing the CB_2_R expression profile, as well as the associated pharmacokinetic and pharmacodynamic aspects of IC patients, are also necessary next steps for optimizing this novel treatment approach.

## 4. Materials and Methods

All animals used in this experiment were obtained from Charles River Laboratories, Inc. (Wilmington, MA, USA) and were acclimatized for 1 week prior to the beginning of experiments. All experimental procedures were performed according to the Canadian Council for Animal Care guidelines and were approved by the Dalhousie University Committee on Laboratory Animals. Animals were housed in the Carlton Animal Care Facility of the Faculty of Medicine at Dalhousie University (Halifax, NS, Canada), were maintained on a light/dark cycle (07:00–19:00), and provided with water and rodent chow *ad libitum*.

### 4.1. Animal Models

#### 4.1.1. IC Induced by i.p. LPS Administration

Female CD-1 mice (30 ± 3 g) were used for pilot IC experiments. IC was induced via i.p. injection of LPS (20 mg/kg from *Escherichia coli* (serotype 026:B6, Sigma-Aldrich, Oakville, ON, Canada) dissolved in 50 μL normal saline (Hospira, Montreal, Canada)). LPS was administered 15 min following induction of anesthesia, and the treatment compounds or vehicle (50 μL) administered i.p. 30 min following LPS administration. The four experimental groups for this model were as follows: (1) healthy control animals (CON; 50 μL normal saline i.p., treated with normal saline i.p., *n* = 9), (2) untreated IC animals (LPS; 50 μL LPS i.p., treated with normal saline i.p., *n* = 9), (3) IC treated with HU308 (LPS + HU308; 50 μL LPS i.p., treated with 5 mg/kg HU308 (Tocris Bioscience, Ellisville, MO, USA) dissolved in 30% DMSO, *n* = 4), (4) IC treated with BCP (LPS + BCP; 50 μL of LPS i.p., treated with 100 mg/kg i.p. BCP (Sigma-Aldrich, Oakville, ON, Canada) in normal saline; *n* = 7). Intravital microscopy (IVM) data was collected two hours following LPS administration for all animals used in this model.

#### 4.1.2. IC Induced by Intravesical LPS Administration with Intravesical Treatment

Female BALB/c mice (20 ± 3 g) were used in this model. IC was induced via intravesical instillation of LPS (150 μg/mL from *Escherichia coli*, dissolved in 50 μL normal saline). LPS was administered 15 min following induction of anesthesia, and the treatment compounds or vehicle (50 μL intravesical volume) administered intravesically 30 min following LPS administration. The five experimental groups for this model were as follows: (1) healthy control animals (CON; 50 μL normal saline, treated with normal saline, *n* = 6), (2) untreated IC animals (LPS; 50 μL LPS, treated with normal saline, *n* = 5), (3) IC treated with 50% DMSO (LPS + 50% DMSO; 50 μL LPS, treated with DMSO 50% (Sigma-Aldrich, St. Louis, MO, USA), *n* = 5), (4) IC treated with BCP (LPS + BCP; 50 μL LPS, treated with 100 mg/kg BCP in saline, *n* = 6). IVM data was collected for all animals used in this model two hours following LPS administration.

#### 4.1.3. IC Induced by Intravesical LPS Administration with Oral Treatment

Female BALB/c mice (20 ± 3 g) were used for this model. IC was induced via intravesical instillation of LPS (150 μg/mL from *Escherichia coli*, dissolved in 50 μL normal saline). LPS was administered 15 min following induction of anesthesia, and the treatment compounds or vehicle administered via gavage one hour prior to LPS administration. The four experimental groups for this model were as follows: (1) healthy control animals (CON; 50 μL intravesical normal saline, treated with oral olive oil, *n* = 5), (2) untreated IC animals (LPS; 50 μL intravesical LPS, treated with oral olive oil, *n* = 5), (3) IC treated with oral BCP (LPS + BCP; 50 μL intravesical LPS, treated with 100 mg/kg BCP dissolved in olive oil, *n* = 5), (4) healthy animals administered oral BCP (BCP; 50 μL intravesical normal saline, treated with 100 mg/kg BCP in olive oil, *n* = 5). IVM data was collected for all animals used in this model two hours following LPS administration. A separate subset of animals from this model was used for behavioral and pain assessments. 

### 4.2. Anesthesia and Surgery

Animals were anesthetized via i.p injection of sodium pentobarbital (65 mg/kg, Ceva Santé Animale, Montreal, QC, Canada). Following induction of anesthesia, mice were placed on a heating pad to maintain a body temperature of 37 °C which was monitored with a rectal temperature probe. Anesthesia was maintained with repeated i.p. administration of 5 mg/kg sodium pentobarbital, while the depth of anesthesia was monitored by clinical examination (return of pedal withdrawal reflex). Once the animals achieved a surgical depth of anesthesia, a Maylard incision of the lower abdomen was performed using surgical scissors. The abdominal muscle layer was lifted using forceps, and an incision made along the linea alba to expose the bladder. Using saline-soaked cotton-tipped applicators, the bladder was gently exteriorized. Credé’s maneuver was then performed to manually void urine from the bladder. Intravesical LPS and treatments were then administered according to the experimental model, as previously described. LPS remained in the bladder for 30 min (for intravesical delivery models) to induce inflammation and was removed by Credé’s maneuver. Immediately after, the treatment compounds were administered and remained in the bladder for the remained of the procedure. IVM was performed two hours following LPS administration.

### 4.3. Intravital Microscopy

In the absence of a toe-pinch reflex, a tail vein injection of two fluorochrome dyes was performed 15 min prior to the start of IVM. The fluorochrome dye mixture consisted of Rhodamine 6G (1.5 mL/kg, 0.75 mg/kg body weight, Sigma-Aldrich, ON, Canada) and fluorescein isothiocyanate (FITC)-albumin (1 mL/kg, 50 mg/kg, Sigma-Aldrich, ON, Canada). With the needle bevel of a 29G x ½ inch 0.5 mL U-100 insulin syringe (BD Canada, reference code: 324703) facing upwards parallel to the vein, the fluorochrome dyes were injected into the tail vein. Rhodamine 6G allows for visualization of leukocytes, while FITC-albumin was used to facilitate evaluation of functional capillary density by providing enhanced illumination of the bladder capillary beds. All tail vein injections were carried out in minimum light to minimize the photobleaching of fluorochromes. Additionally, the fluorochrome solutions were stored in foil-covered receptacles.

After the 15-min period, a small clean glass cover slip was positioned on top of the bladder, and the animal positioned under the microscope. To avoid movement of the bladder as a result of diaphragm activity, a metal arm was used to apply gentle pressure to the upper abdomen. Any areas of the abdomen that were not subject to experimentation were covered in gauze that was saturated in saline and maintained at physiological temperature to avoid dehydration and exposure to ambient air. Intravital fluorescent video microscopy was performed using the following technical devices: an epifluorescence microscope (Leica DMLM, Wetzlar, Germany, Filterset: I3, green light filter), light source (LEG EBQ 100, Jena, Germany), and a black and white monitor (Speco Technologies, Texas, US). The images were transferred to a Windows desktop computer and recorded using WinDV software (version 1.2.3, Czech Republic). The leukocytes within the microcirculation of the bladder were visible under the 20× objective with green light. Five to seven randomly selected visual fields containing bladder venules were recorded for 30 s. The filter was then changed for examination with FITC-albumin under blue light, allowing for the examination of capillary blood flow. Five to seven randomly selected visual fields with capillaries were recorded again for 30 s.

### 4.4. Video Analysis

Assessment of microcirculatory parameters collected from IVM was performed off-line by analysis of recorded videos using ImageJ software (National Institute of Health, USA). We analyzed two microcirculatory parameters; leukocyte adhesion and functional capillary density. When examining leukocyte adhesion, the length and diameter of the venule in the field of view was used to calculate the endothelial surface area. Adherent leukocytes were defined as white blood cells that stayed immobile on the endothelial surface for the complete observation period of 30 s. The number of adherent leukocytes in a randomly selected vessel was reported as number of cells per square millimeter of endothelial surface area. When analyzing FCD, for each video a single rectangular field covering the maximum possible area was drawn, and capillaries containing FITC-albumin marked plasma found in the rectangular field were chosen. The length of perfused capillaries was measured by manually drawing a line within the lumen of the capillary. Capillaries with absent and/or intermittent flow were characterized as dysfunctional capillaries and not accounted for, whereas capillaries with continuous flow, regardless of the speed of the flowing cells, were characterized as functional capillaries. Summing the length of all corresponding capillaries and dividing the sum length with the measured area of the rectangular field calculated the FCD. For the initial pilot segment of this study, FCD was expressed as a percentage relative to the FCD of the control group.

### 4.5. Behavior Assessment Scoring

Prior to LPS and/or treatment administration, all animals were observed and scored to set an individual baseline score for each animal. Animals were then anesthetized with isoflurane and administered saline or LPS intravesically, according to the group. For animals receiving oral BCP, the treatment was administered by gavage 60 min prior to saline or LPS instillation. Following the instillation, animals were woken up in their cages and allowed to explore and feed *ad libitum* under observation for one hour. Changes in animal behavior and appearance (i.e., breathing rate, posture, motor activity, eye opening) were then scored again. Each behavioral parameter can receive a maximum score of 10 (worst case), summing to a maximum cumulative score of 40. A minimum score of 0 implies that the animals show no signs of pain. Animals in pain typically exhibit the following; increased breathing rate, fully or partially closed eyes, hunched posture and overall less motor activity. The behavioral scoring system was adapted from Boucher et al. 2000 [59] (Table 1).

### 4.6. Von Frey Aesthesiometry

Electric von Frey aesthesiometry (IITC Inc. Life Science 2390 series, Woodland Hills, California, USA) was performed to assess pain tolerance by measuring the amount of force applied (in grams) before the animal reacted (e.g., withdraw) to a noxious stimulus directed at the suprapubic region of the lower abdomen. Healthy animals were scored prior to IC induction to set a baseline for each individual animal, as done in the behavioral assessment scoring. Animals were allowed to acclimate in the procedure room for 1 h prior to baseline assessment. The lower abdomen of the animal was then shaved, the suprapubic region marked with a marker, and LPS instilled into the bladder. Animals were allowed to acclimate in the procedure room for two hours following LPS instillation. Behavioral data were collected following the first hour of this acclimation period. The animal was then placed in a plexiglass enclosure with a mesh floor (IITC Life Sciences, Woodland Hills, CA, USA) and allowed to acclimatize for 15 min. Following the acclimation period, a rigid tip probe attached to the aesthesiometer was applied to the marked spot of the abdomen through the mesh floor, until a withdrawal was noted. Five values were recorded for each animal, with a minimum 30-s interval between measurements. Each animal was compared to itself before the induction and following treatment, and a summarized group comparison was also performed.

### 4.7. Statistical Analysis

All data are expressed as means ± standard deviation (SD), except for the FCD measurements in the IC model induced by i.p. LPS, which is expressed as a percentage relative to the control group. Statistical analyses of the results were performed using the software GraphPad Prism 6.0 (GraphPad Software Inc, La Jolla, CA, USA). After confirmation of normal distribution by Kolmogorov-Smirnov testing, differences between groups were analyzed using one-way ANOVA, followed by Tukey’s multiple comparison test for group wise comparisons. The significance level was considered at *p* < 0.05.

## Figures and Tables

**Table 1:** Description of parameters used in behavior scoring system.

Parameter	Score Description
Breathing Rate	Each decrease/increase of 10 breaths per minute from baseline warrants an increase/decrease in score.
Eye opening/closing	0: eyes completely open 5: eyes half closed 10: eyes fully closed
Posture	0: normal posture, 5: moderately hunched back but able to stretch for food/water, 10: fully rounded back or limp posture, no attempt to reach for water or food. Intermediate scores were assigned based on the discretion of observer.
Motor activity	Activity (exploring, grooming, feeding) within 20 s (Ex. No movement for 10 s (=50%) = 5 points, no movement for 10 s (=10%) = 1 point

## Supplementary Materials

The following are available online. Figure S1: Leukocyte adhesion in submucosal bladder venules of female wild-type and CB_2_R knockout (KO) BALB/c mice for the following experimental groups: wildtype control (CON, *n* = 5), LPS-induced IC in wildtype animals (LPS, *n* = 5), CB_2_R KO control (KO-CON, *n* = 5), LPS-induced IC in CB_2_R KO (KO-LPS, *n* = 4). LPS groups received a 50 μL intravesical instillation of 150μg/mL LPS, which was replaced by 50 μL of saline after 30 min. Control groups received 50 μL saline intravesically, replaced by another 50 μL saline instillation after 30 min. IVM was performed at the end of the 2 h observation time. Data is presented as mean ± SD. * < 0.05.
