# Supplementary material for: Experimental Cannabinoid 2 Receptor Activation by Phyto-Derived and Synthetic Cannabinoid Ligands in LPS-Induced Interstitial Cystitis in Mice
Source: Molecules. 2019 Nov 21;24(23):4239. doi: 10.3390/molecules24234239 (PMC6930590; doi:10.3390/molecules24234239)
Supplement: Supplementary file 1 [file molecules-24-04239-s001.pdf]

Supplementary Materials:

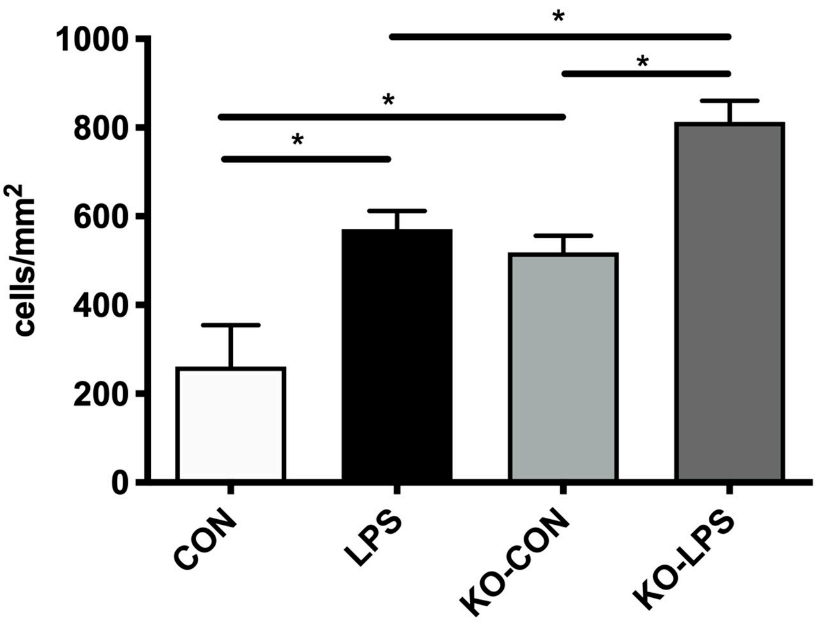

Figure S1. Leukocyte adhesion in submucosal bladder venules of female wild-type and CB_2R knockout (KO) BALB/c mice for the following experimental groups: wildtype control (CON, $n = 5$), LPS-induced IC in wildtype animals (LPS, $n = 5$), CB_2R KO control (KO-CON, $n = 5$), LPS-induced IC in CB_2R KO (KO-LPS, $n = 4$). LPS groups received a 50 μL intravesical instillation of 150 μg/mL LPS, which was replaced by 50 μL of saline after 30 min. Control groups received 50 μL saline intravesically, replaced by another 50 μL saline instillation after 30 min. IVM was performed at the end of the 2 h observation time. Data is presented as mean ± SD. * < 0.05.
